# Anticancer Small-Molecule Agents Targeting Eukaryotic Elongation Factor 1A: State of the Art

**DOI:** 10.3390/ijms24065184

**Published:** 2023-03-08

**Authors:** Han Zhang, Jiayou Cai, Siqi Yu, Bin Sun, Weicheng Zhang

**Affiliations:** The State Key Laboratory of Medicinal Chemical Biology, College of Pharmacy, and Tianjin Key Laboratory of Molecular Drug Research, Nankai University, Tianjin 300353, China

**Keywords:** anticancer, eEF1A, mechanism, macrocycle, heterocycle

## Abstract

Eukaryotic elongation factor 1A (eEF1A) canonically delivers amino acyl tRNA to the ribosomal A site during the elongation stage of protein biosynthesis. Yet paradoxically, the oncogenic nature of this instrumental protein has long been recognized. Consistently, eEF1A has proven to be targeted by a wide assortment of small molecules with excellent anticancer activity, among which plitidepsin has been granted approval for the treatment of multiple myeloma. Meanwhile, metarrestin is currently under clinical development for metastatic cancers. Bearing these exciting advances in mind, it would be desirable to present a systematic up-to-date account of the title topic, which, to the best of our knowledge, has thus far been unavailable in the literature. The present review summarizes recent advances in eEF1A-targeting anticancer agents, both naturally occurring and synthetically crafted, with regard to their discovery or design, target identification, structure–activity relationship, and mode of action. Their structural diversity and differential eEF1A-targeting mechanisms warrant continuing research in pursuit of curing eEF1A-driven malignancy.

## 1. Introduction

The majority of cancers arise from accumulated somatic mutations, which over time, transform the cell into a state of malignancy. This is characterized by uncontrolled proliferation, aggressive invasion into surrounding normal tissues, and ultimately lethal metastasis at distant vital organs. Genomic instability and phenotypic heterogeneity inherent in each malignant tissue [1] render the treatment of cancer extremely challenging [2]. Today, apart from surgical operation, a variety of promising therapeutic strategies have been developed, including but not limited to radiotherapy [3], chemotherapy [4], biological therapy [5], immunotherapy [6], and microbial-based therapy [7,8]. From this ever-growing curative armamentarium, targeted chemotherapy with small-molecule or biomacromolecular agents is an indispensable measure [9,10,11].

Eukaryotic elongation factor 1A (eEF1A, formerly termed eEF-1α) is an essential GTPase evolutionarily conserved across diverse eukaryotes [12]. As the second most abundant intracellular protein after actin, it is localized extensively in the cytoplasm and nucleus [13,14,15]. The canonical function of eEF1A is to deliver amino acyl tRNAs to the ribosomal A site during the elongation stage of protein synthesis [16]. Strikingly, beyond this housekeeping role for the translational machinery and many other moonlighting functions [17,18,19,20], mounting evidence has pointed to a causal link between eEF1A and malignancy [21,22,23], suggesting that eEF1A may serve as not only an out-of-control translational cofactor [24,25,26] but also a signal transducer woven into a network of protumorigenic pathways [27,28,29,30,31,32]. Consistent with this revelation, structurally distinct small-molecule anticancer agents continue to emerge with a proven eEF1A-targeting mechanism [33]. Early examples include didemnin B [34], plitidepsin (dehydrodidemnin B) [35], tamandarin A [36], cytotrienin A [37], ansatrienin B [38], narciclasine [39], and synthetic flavonoids [40]. Among them, plitidepsin was approved in Australia for combined treatment of relapsed/refractory multiple myeloma with dexamethasone [41], thus providing initial proof of principle that eEF1A inhibitors can achieve the desired therapeutic efficacy with safety. In recent years, more intriguing compounds of this kind have been discovered and actively investigated. As a notable case, metarrestin is currently in a phase I clinical trial for the treatment of metastatic solid tumors [42]. Though a number of reviews have been published on protein-synthesis inhibitors [43,44,45,46], to the best of our knowledge, no systematic survey has ever been conducted on eEF1A-targeting agents. Hence, the present review delineates the state of the art on eEF1A-targeting small-molecule anticancer agents with a special focus on those actively studied over the recent years, covering their discovery or design, target identification, structure–activity relationship (SAR), and mode of action.

## 2. Recent Advances in Anticancer eEF1A-Targeting Agents

### 2.1. Didemnins and Tamandarins

**Figure 1:**
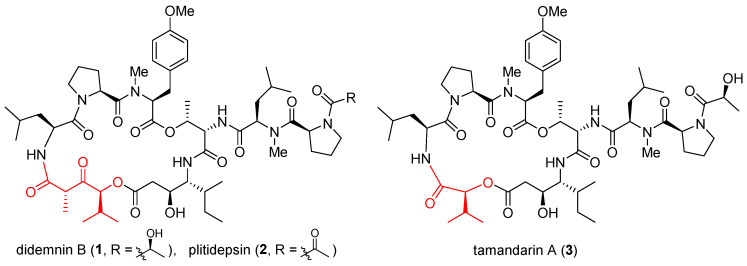
Structures of didemnin B (**1**), plitidepsin (**2**), and tamandarin A (**3**).

Didemnins are a family of marine cyclic depsipeptides with strong anticancer, antiviral, and immunosuppressive activities [47]. Since their initial discovery in the early 1980s [48], these macrocycles have become the subject of intense research over the last four decades. Among them, didemnin B (**1**, Figure 1) and plitidepsin (**2**) have entered multiple clinical trials. Gratifyingly, plitidepsin was approved in Australia for treating multiple myeloma [41]. With nearly identical architectures but subtly different macrocyclic backbones (highlighted red), tamandarins such as tamandarin A (**3**) were discovered from a different colony of marine ascidian [49]. As already reviewed in multiple comprehensive monographs [36,50,51,52,53,54,55,56], these compounds will not be reiterated here. Instead, a brief update is presented below on their anticancer mode of action.

Having developed functional signature ontology (FUSION) maps for drug discovery and mechanistic elucidation [57], White et al. revisited the mechanism of **1** and found that it induces rapid and extensive apoptosis in sensitive cancer cell lines through concomitant inhibition of palmitoyl-protein thioesterase 1 (PPT1) and eEF1A1 [58]. Independently, Galmarini et al. showed that eEF1A2 is the specific binding target of **2** with a measured dissociation constant (*K*_D_) of 80 nM [35]. Since translation inhibition cannot account per se for the observed antiproliferative effect of **2**, it was suspected that this drug impacts non-canonical functions of eEF1A2. Indeed, double-stranded RNA (dsRNA)-dependent protein kinase (PKR) was later identified as a novel binding partner of eEF1A2 [30]. In this case, eEF1A2 interacts directly with PKR to block its pro-apoptotic activity and boost tumor survival. In the presence of **2**, however, PKR was disengaged from eEF1A2, thereby regaining its kinase activity to initiate extrinsic apoptosis through activation of MAPK and NF-κB signaling cascades [30]. More recently, Martinez-Leal et al. reported that **2** induces endoplasmic reticulum (ER) stress in HeLa cells by activating the multipronged unfolded protein response (UPR) in a characteristic pattern [59]. Working simultaneously as an ER stress inducer and an autophagy inhibitor, **2** was combined with bortezomib to synergistically block proteasomal degradation and autophagy, thereby exacerbating accumulation of misfolded proteins that originate from plitidepsin-induced oxidative stress. Such elevated proteotoxic stress led to apoptosis both in vitro (in MM1S multiple myeloma cells) and in vivo (in SCID mice xenografted with RPMI-8226 multiple myeloma cells). This study shows the promise of combined anticancer therapy using plitidepsin and proteasomal inhibitors such as bortezomib in a clinical setting.

### 2.2. Cytotrienin A and Ansatrienin B

**Figure 2:**
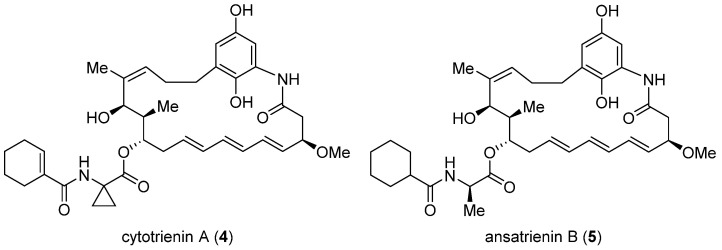
Structures of cytotrienin A (**4**) and ansatrienin B (**5**).

Cytotrienin A (**4**, Figure 2) was initially isolated from the culture broth of soil-dwelling *Streptomyces* sp. RK95-74 [60]; this compound has strong cytotoxicity (IC_50_ = 7.7 nM) against human leukemia cell line HL-60 [61]. Despite early mechanistic studies [62,63,64], its target remained elusive until Pelletier et al. identified **4** as a translation inhibitor through high-throughput screening [37]. Their finding is that akin to didemnin B, cytotrienin A modulates eEF1A-dependent loading of aa-tRNA to the ribosome, most likely by stabilizing the eEF1A/GTP/aa-tRNA assembly positioned at the ribosomal A site. Thus, without release of eEF1A from the ribosome, translation elongation stops. Further insight into this compound’s mode of action came from a 2015 study led by Taunton, who aimed to seek out the target of a potent antiproliferative ternatin derivative **7** (ternatin-4, cross-refer to the following section) [38]. With the help of photoaffinity labeling, they were able to capture the binding partner with a ternatin-based probe. Interestingly, the photolabeled protein is a ternary complex comprising eEF1A, GTP, and aa-tRNA rather than eEF1A alone. Subsequent competitive-binding experiments noted that the photolabeling efficiency is diminished dose-dependently with the addition of didemnin B or ansatrienin B (**5**, a close side-chain analogue of cytotrienin A shown in Figure 2). Therefore, it was concluded that ternatin, didemnin B, and cytotrienin A/ansatrienin B may share a binding hot spot on the eEF1A surface, probably located near A399, as indicated by resistance-conferring mutation experiments.

### 2.3. Ternatin-4

**Figure 3:**
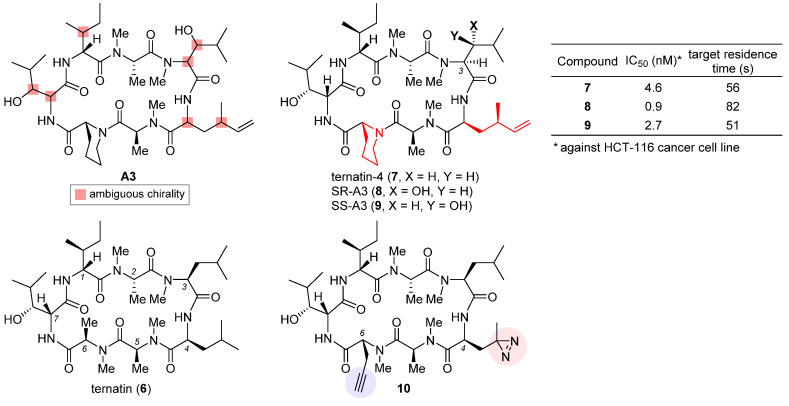
Structures of natural products **A3** and ternatin (**6**), and synthetic analogues ternatin-4 (**7**), SR-A3 (**8**), SS-A3 (**9**), and photoaffinity probe **10**. The inset table compares anticancer activity and target residence time of **7–9**.

The highly cytotoxic natural product **A3**, together with several other congeners, was isolated from an *Aspergillus* fungus [65]. Although its structure was determined to be a partially *N*-methylated cyclic heptapeptide, the chirality of 7 out of 11 stereo-centers (marked in the structure of **A3**, Figure 3) remained unknown. The strong structural similarity between **A3** and ternatin (**6**, CAS registry number: 148619-41-4) [66], another natural product with anti-obesity activity [67], inspired Taunton et al. to design ternatin-4 (**7**) by incorporating the dehydromethyl leucine and pipecolic acid residues of **A3** (highlighted red in the structure of **7**, Figure 3) into **6 [38]**. The resulting hybrid molecule **7** attained more than 10-fold enhancement of potency over the parent compound **6** (IC_50_ 4.6 nM vs. 71 nM against HCT-116 cancer cell line), thus solving all but one stereo-configuration of **A3**. Wondering the molecular target of ternatins, they developed a bifunctional photoaffinity probe **10**. Under UV irradiation, its photolabile diazirine subunit at residue 4 (highlighted red) decomposes into a highly reactive carbene that instantaneously crosslinks to the nearby binding protein. The alkyne at residue 6 (highlighted blue) will then connect to a fluorescent reporter via click cycloaddition, thus tagging the photolabeled target for characterization. In this way, eEF1A was captured and confirmed as the target of ternatins.

Based on the verified structure of ternatin-4 (**7**), a β-hydroxyl group was introduced at its residue 3 to obtain two epimers of **A3**, namely SR-A3 (**8**) and SS-A3 (**9**). An improved second-generation total synthesis allowed quick access to both compounds [68]. Hence, the identity of natural product **A3** was established as SR-A3 (**8**). Of special note, despite a minor difference in the side chain of residue 3, **8** was found to display a more prolonged duration of growth inhibition action than **7** and **9**. Single-molecule fluorescence resonance energy transfer (smFRET) imaging corroborated this observation, while further quantification through in vitro chase experiments confirmed enhanced drug–target residence time (table inset in Figure 3) and rebinding kinetics of **8**. Finally, preclinical evaluation of **8** vis-a-vis **7** was carried out in an aggressive Myc-driven mouse lymphoma model. Compared with its des-hydroxyl variant ternatin-4 (**7**), SR-A3 (**8**) significantly reduced tumor burden while extending the survival of the treated Eμ-Myc mice [68]. This work highlights the importance of side-chain modification in macrocyclic drug discovery and also makes a good case that the drug–target interaction can be more precisely characterized using the drug–target residence time model [69]. More recently, with the help of smFRET imaging and cryogenic electron microscopy (cryo-EM), Taunton and collaborators demonstrated that in spite of sharing a common eEF1A-binding site, ternatin-4 (**7**) and didemnin B exhibit differential inhibition dynamics in that the former traps the eEF1A/GDP/aa-tRNA ternary complex on the ribosome in a more reversible fashion than does the latter [70]. Their in-depth mechanistic investigation also revealed that by trapping eEF1A at the ribosomal A site, ternatin-4 induces ubiquitination and degradation of eEF1A on stalled ribosomes through a previously unknown surveillance pathway for translation quality control [71].

### 2.4. Nannocystin A

**Figure 4:**
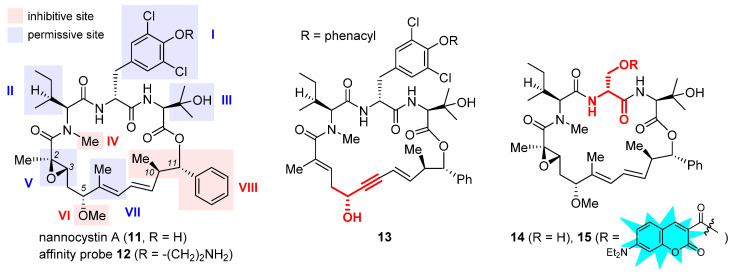
Structures of naturally occurring nannocystin A (**11**), semisynthetic affinity probe **12**, as well as synthetic macrocyclic propargylic alcohol **13**, serine-containing intermediate **14**, and coumarin-tagged fluorescent probe **15**. The SAR illustrated in the structure of **11** consists of inhibitive (red) and permissive (blue) sites: the former must be reserved for high activity, whereas the latter can tolerate moderate change.

Nannocystin A (**11**, Figure 4) is a 21-membered cyclic depsipeptide isolated independently by Brönstrup et al. [72] and Hoepfner et al. [73] from the myxobacteria of the *Nannocystis* genus. Brönstrup et al. found that **11** is a strong inducer of apoptosis, as such inhibiting the growth of multiple cancer cell lines with low nanomolar IC_50_ values [72]. Meanwhile, another team led by Hoepfner pinned down its target through a combination of genetic and chemoproteomic approaches [73]. In brief, initial haploinsufficiency profiling and mutagenesis experiments implied eEF1A as the most likely target. To verify direct binding, they set up affinity chromatography with the semisynthetic probe **12** (Figure 4). During elution, this immobilized nannocystin sequestered eEF1A1 and eEF1A2 out of the 3644 proteins comprising the HCT-116 cell lysates. Moreover, it competed with unbound nannocystin A (**11**) and didemnin B for binding to eEF1A. Hence, eEF1A was determined to be the target of nannocystin A [73].

The firsthand structure–activity relationship of nannocystins was derived from isolated and semisynthetic nannocystins [72,73], which indicates that modification at the tyrosine phenol moiety (subdomain **I** in the structure of **11**, Figure 4) or the side chain of *N*-Me-L-isoleucine (subdomain **II**) is tolerated. To obtain comprehensive SAR, nevertheless, total synthesis is a must. Thus far, seven routes have been reported for the total syntheses of nannocystin A (**11**) or its 2*E*-alkene surrogate nannocystin Ax (**16**, structure shown in Table 1) [74,75,76,77,78,79,80,81], each involving a distinct macrocyclization reaction as the key strategic step [82]. With the dual purpose of (1) total synthesis and (2) SAR validation concerning the binding role of the polyketide C5-C7 region, Fürstner et al. devised a motif-oriented strategy so that the macrocyclic propargylic alcohol **13** (shown in Figure 4) underwent post-macrocyclization elaboration [83] into an array of novel analogues besides nannocystin Ax (**16**) [80]. It was found that the 5*R*-methoxy ether (subdomain **VI**), instead of the neighboring C6-C7 (*E*)-alkene (subdomain **VII**), must be reserved for high activity. As an illustration, Table 1 compares the anticancer activity of four pairs of nannocystin derivatives with or without methylation at the C5-OH group (R = Me or H). Clearly, removal of this moiety causes a drastic reduction in potency (**16** vs. **17**, **18** vs. **19**, **20** vs. **21**, **22** vs. **23**); on the other hand, changing the C6-methyl (**16**) to a fluorine (**18**) or hydrogen (**20**) atom, or even curtailing the (*Z*) alkene to an alkyne (**22**), has insignificant impact on activity.

Following a total synthesis of nannocystin A [76] via Heck macrocyclization [84,85], we prepared a diversity of non-natural nannocystins modified at different sites. Our findings demonstrated that (1) the (2*R*, 3*S*)-epoxide (subdomain **V**) may be substituted for a 2*E*-alkene without compromising activity [86], (2) the side chain of β-OH-L-valine (subdomain **III**) is tolerant of minor change [86], and (3) the polyketide C9-C10 segment including its entire (10*R*, 11*S*) stereo-chemistry (subdomain **VIII**) is a key determinant of potency [87,88]. In parallel, He et al. synthesized more variants via Heck macrocyclization too and observed that removal of the *N*-methyl moiety (subdomain **IV**) incurred a dramatic loss of activity [89]. Taken together, the SAR of nannocystins is illustrated in the structure of **11** (Figure 4).

Aside from target elucidation and SAR profiling, the exact mechanism of nannocystins is a subject of enduring interest [90,91,92] given the poorly understood role of eEF1A in tumorigenesis. Chen et al. showed that the antimetastatic effect of nannocystin Ax in lung cancer cells is attributable to its interference with the TGF-β/Smad signaling pathway [90]. Their result is in step with another study uncovering the promigratory ability of eEF1A2 to promote lung adenocarcinoma metastasis [32]. The additional finding that the regulation of TGFβI (TGF β receptor I) by nannocystin Ax occurs at the transcriptional rather than the (post-)translational level implied the presence of an alternative mechanism independent of eEF1A inhibition [90]. Therefore, similar to the case of plitidepsin [30], the possibility that nannocystins impact certain protumorigenic pathway(s) mediated by eEF1A cannot be ruled out. Recently, we designed a serine-incorporating nannocystin **14** (Figure 4) to leverage a post-macrocyclization diversification strategy for efficient side-chain variation [92]. Thus obtained SAR concurred with the general trend depicted in Figure 4 and further informed the development of a coumarin-conjugated fluorescent probe **15**. With good permeability into the cancer cells, this probe was localized to the ER, as visualized by confocal fluorescence microscopy, which implies that nannocystins act on eEF1A predominantly at the ER-bound ribosome. Our result is in good agreement with the latest work by Förster et al. capturing eEF1A associated with the ribosome at the ER membrane by the use of cryo-electron tomography [93], thereby shedding light on the intracellular mode of action of nannocystins.

### 2.5. Metarrestin

Perinucleolar compartment (PNC) is a heritable multicomponent dynamic subnuclear organelle located at the periphery of the nucleolus of eukaryotic cells and uniquely associated with metastatic cancer cells [94]. Huang et al. found that PNC prevalence, defined as the percentage of non-apoptotic and non-mitotic cells harboring at least one PNC, is a pan-cancer prognostic marker positively correlated with metastatic capacity [95,96]. Subsequent screening of clinically approved anticancer drugs led to the observation that some of these drugs are capable of reducing PNC prevalence via specific on-target inhibition in lieu of promiscuous toxicity [97]. Having confirmed the existence of mechanism-specific PNC disassemblers with clinical efficacy, this proof-of-concept study supported taking PNC prevalence reduction as a phenotypic screening marker to discover antimetastatic drugs. To this end, a metastatic prostate cancer cell line PC3M with a PNC prevalence of 75% to 85% was engineered to stably express green fluorescent protein (GFP)-fused polypyrimidine-tract-binding protein (PTB) [98]. PTB is an essential PNC marker routinely measured by immunohistochemistry, which is unfortunately inconvenient for automated screening. But now with the self-reporting fluorescent PC3M-GFP-PTB cell line at hand, they were able to establish an image-based high-throughput, high-content assay (HCA) primed for spotting compounds able to reduce PNC prevalence by 50% [99]. Aiming at antimetastasis, the initial hits underwent secondary assays to select for invasion inhibition while excluding those acting via apoptosis induction, DNA intercalation, general cytotoxicity, or cell-cycle arrest [100]. By means of this multistage screening protocol, two leads were eventually identified out of 140,800 structurally diverse compounds from the NIH Molecular Libraries Small Molecule Repository (MLSMR) due to their outstanding PNC-disassembling efficiency and low cytotoxicity, thus setting the stage for the ensuing medicinal chemistry campaign [101].

**Figure 5:**
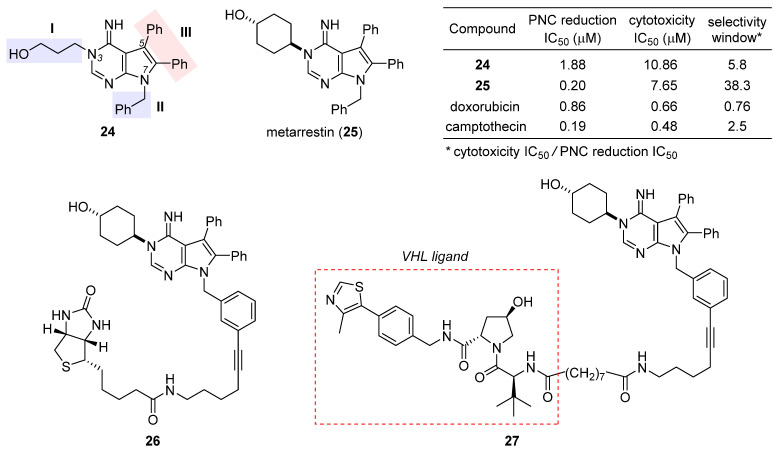
Structures of the initial hit **24**, final drug candidate metarrestin (**25**), biotin-labeled affinity probe **26**, and metarrestin-based PROTAC **27**. The inset table compares the selectivity window between PNC reduction and cytotoxicity of **24**, **25**, doxorubicin, and camptothecin.

After a preliminary exploration, pyrrolopyrimidine **24** (Figure 5) was favored over the other lead (structure not shown) for systematic optimization. Robust synthetic methods were next developed to access a broad variety of analogues evaluated for PNC disassembly and drug-like properties as well. The SAR trends are summarized in the structure of **24** (Figure 5). Specifically, (1) the *N*-3 substitution at the subdomain **I** prefers a linear alkyl chain bearing a hydroxy, ether, or amine, and conformational constraint with a cyclohexyl ring gives rise to the highest potency; (2) the *N*-7 position at the subdomain **II** tolerates a benzyl, phenethyl, or 4-methoxylphenyl group, but the presence of an alkyl substituent diminishes the potency significantly; (3) the unsubstituted C5 and C6 phenyl rings at the subdomain **III** are indispensable for high potency. While deducing the above trends, multi-round optimization finally yielded metarrestin (**25**), which possesses a superior selectivity window between PNC reduction and cell viability compared with the classic anticancer drugs doxorubicin and camptothecin (table inset in Figure 5) [101]. The in vitro performance of **25** was smoothly translated into in vivo efficacy in three mouse models of human cancer, where it suppressed metastatic invasion with concomitant reduction in PNC prevalence in the cancer cells of primary and metastasized tumors, offering a remarkable survival advantage to the treated animals [100]. After an in-depth evaluation of its pharmacokinetics [102,103] and safety [104], this drug has been advanced into a phase I clinical trial for the treatment of metastatic solid tumors [42].

The excellent antimetastatic capability of **25** prompted Huang et al. to investigate its mechanism, with the primary conclusion that the drug disrupts PNC assembly by blocking RNA polymerase I transcription [100]. Further seeking the binding target of **25**, they designed a biotin-conjugated probe **26** (Figure 5) that is likewise efficacious in disassembling PNC. Affinity purification with **26** combined with competition experiments using untagged metarrestin identified eEF1A2 as the binding target. The metarrestin–eEF1A2 interaction was confirmed by cellular thermal shift assay. Subsequent experiments along this line of research showed that (1) eEF1A2 enhances PNC assembly and metastatic progression; and (2) eEF1A2, at least in part, mediates the PNC-elimination function of metarrestin [100]. Whereas further details await elucidation, it was believed that metarrestin interferes with certain non-translational functions of eEF1A2. Recently, Jin et al. developed a proteolysis-targeting chimera (PROTAC) [105,106,107] strategy by tethering metarrestin with various ligands for the von Hippel–Lindau (VHL) E3 ligase [108]. Thus obtained heterobifunctional molecules were designed to recruit eEF1A2, the binding target of metarrestin, to the ubiquitin/proteasome system (UPS) for selective degradation. As one of these first-in-class eEF1A2 degraders, **27** (Figure 5) was shown to degrade eEF1A2 in three cancer cells in a dose-dependent manner, thus holding promise for the treatment of eEF1A2-mediated carcinogenesis.

### 2.6. 2-Phenyl-3-Hydroxy-4(1H)-Quinolinones

**Figure 6:**
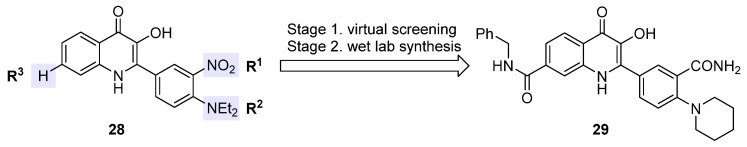
Structures of eEF1A1-targeting anticancer 3-HQ derivative **29** and its prototype **28**.

2-Phenyl-3-hydroxy-4(1*H*)-quinolinones (abbreviated as 3-HQs) such as **28** (Figure 6) are aza-analogues of previously reported eEF1A-targeting anticancer flavonoids [40]. Based on a homology model of human eEF1A1, Hlavac et al. carried out docking calculations to identify the binding site for gamendazole, a known eEF1A1 inhibitor for male contraception [109]. Encouragingly, they found that these 3-HQs fit into the same gamendazole-binding site on the surface of eEF1A1. Such direct interaction between eEF1A1 and 3-HQs was verified through pull-down assay using biotinylated 3-HQ derivatives [110]. Having validated the constructed eEF1A1 model, the authors performed virtual screening of in silico designed 3-HQs with varying substituents R^1^, R^2^, and R^3^ (illustrated in the structure of **28**). The six highest-scored and synthetically accessible compounds were chosen for wet-lab preparation. Their binding to eEF1A1 was quantitatively characterized with isothermal titration calorimetry (ITC), which provided thermodynamic information consistent with docking calculation results. Biological evaluation discovered **29**, one of these rationally designed eEF1A1 inhibitors, with optimal inhibitory activity against several cancer cell lines but low toxicity toward a normal cell line [110].

### 2.7. Cordyheptapeptide A

**Figure 7:**
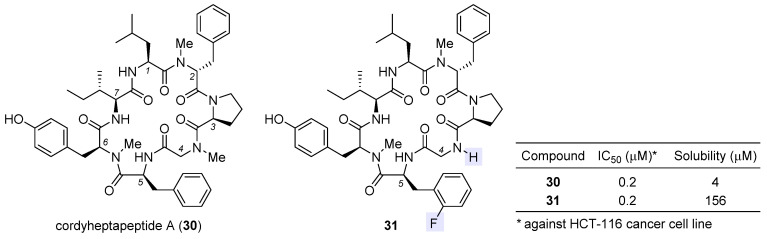
Structures of natural cordyheptapeptide A (**30**) and synthetic analogue **31**, with the inset table comparing their IC_50_ values against HCT-116 cancer cell line and aqueous solubility.

Cordyheptapeptide A (**30**), a partially *N*-methylated cyclic heptapeptide with anticancer activity, was originally isolated from the insect pathogenic fungus *Cordyceps* [111,112]. Although a solution-phase total synthesis of **30** was reported before [113], its SAR and mechanism of action were unclear. Lokey et al. developed a high-throughput solid-phase peptide synthesis (SPPS) to access a library of side-chain- and backbone-modified analogues [114]. They observed the following SAR trends: (1) all side chains are critical to its antiproliferative activity [115]; (2) halogenation at the aromatic side chain of residue 2 or 5 deteriorates activity at varying degrees; (3) whereas removal of the *N*-methyl moiety at residue 2 or 6 impairs activity, this is not the case for residue 4, for which changing sarcosine to glycine tends to improve activity, and when coupled with *ortho*-fluorination at residue 5, such *N*-demethylation brings about equipotent variant **31** with a 39-fold improvement in aqueous solubility (table inset in Figure 7). According to molecular dynamics simulations, the enhancement in activity stems from more conformational flexibility of its glycine-carrying scaffold, which is accordingly more accessible to target-binding conformations than the parent natural product **30**.

To find out the mechanism of action of **30**, the authors determined its cytotoxicity profile via the NCI60 human tumor cell line assay [114]. Analyzed by the COMPARE algorithm, this profile was best correlated with that of phyllanthoside, a known eukaryotic protein-synthesis inhibitor [116]. Consistently, cytological profiling (CP) [117] indicated that **30** clustered most closely with protein-synthesis inhibitors such as didemnin B and ternatin but deviated significantly from microtubule inhibitors and poly (ADP-ribose) polymerase (PARP) inhibitors. Combining the results from both phenotypic experiments, **30** is quite likely a protein-synthesis inhibitor. This inference was confirmed by bioorthogonal noncanonical amino acid tagging (BONCAT) [118], which proved that the agent primarily blocks protein synthesis and has a secondary influence on DNA synthesis. Suspecting its target to be eEF1A, **30** was evaluated in the HCT-116 cancer cells with a point mutation of eEF1A (A399V). Previously, the same mutation was reported to confer resistance to eEF1A-targeting didemnin B [119], ternatin [38], and nannocystin A [73]. Indeed, the activity dropped remarkably in the mutant cells, thus providing genetic evidence that supports eEF1A as the target of **30**.

### 2.8. BE-43547A_2_

**Figure 8:**
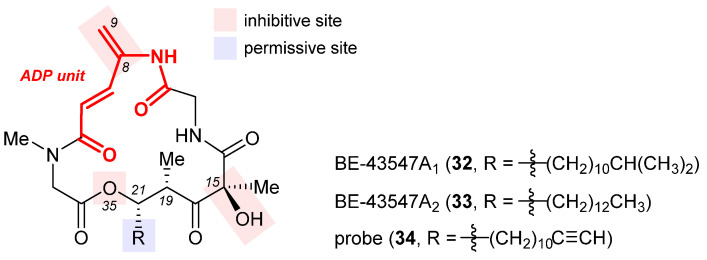
Structures of BE-43547A_1_ (**32**), BE-43547A_2_ (**33**), and the clickable probe **34**. The SAR illustrated in their structures consists of inhibitive (red) and permissive (blue) sites: the former must be reserved for high activity, whereas the latter can tolerate moderate change.

Isolated from *Streptomyces* sp. in 1998, BE-43547A_1_ (**32**, Figure 8), BE-43547A_2_ (**33**), and other congeners are a series of macrocyclic depsipeptides differing in the C21 side chain [120]. These compounds belong to the amidopentadienoate-containing cyclolipodepsipeptide (APD-CLD) natural products that feature an electrophilic 4-amido-2,4-pentadienoate (APD, highlighted red) functionality as well as a lipophilic side chain [121]. Poulsen et al. developed the first total synthesis of *ent*-**32** and re-isolated the authentic **32** from the fermentation broth of a BE-43547-producing microorganism, thereby establishing the absolute stereo-configuration of the BE-43547 family [122]. Importantly, **32** and **33** exhibited superior hypoxia-selective cytotoxicity in PANC-1 pancreatic cancer cells than rakicidin A, another APD-CLD natural product they investigated earlier [123,124,125,126]. Shortly after this work, Chen et al. developed a total synthesis of **33** and reported that this agent selectively targets pancreatic cancer stem cells (PCSCs) [127,128]. Preparation of more analogues [129,130] led to the following SAR results (illustrated in Figure 8): (1) the exocyclic alkene at C8 within the APD unit must be reserved; (2) the macrolide cannot be changed to the corresponding macrolactam, or in other words, the O35 cannot be replaced with a nitrogen atom; (3) the (*S*)-hydroxyl group at C15 is critical to activity; (4) a lipophilic side chain at C21 is necessary but variable.

The excellent hypoxia-selective toxicity of APD-CLD natural products such as rakicidin A and the BE-43547A members intrigued Poulsen et al. to elucidate their mechanism of action, which is distinguished from conventional hypoxia-activated compounds. They showed that these APD-CLDs induced rapid and hypoxia-selective impairment of mitochondrial structure and function, thereby driving a peculiar form of non-apoptotic cancer cell death in a hypoxic milieu [131]. To discover the molecular target of **33**, Chen et al. synthesized a clickable probe **34** [130]. Biotinylation of **34** via in situ click cycloaddition followed by pull-down assay indicated eEF1A as a binding target, and the isoform was determined to be eEF1A1 via immunoblotting. Having located the cysteine234 residue of eEF1A1 as the most probable binding site of **33** according to LC-MS/MS analysis, they engineered three types of pancreatic cancer cells with (1) eEF1A1 knockdown (KD), (2) eEF1A1 recovered from KD (RE-KD), and (3) C234-mutant eEF1A1 constructed from KD (RE-C234S). As shown in Table 2, the in vitro cytotoxicity of **33** against these three and the wild-type (WT) pancreatic cancer cells, as well as its in vivo anticancer efficacy in the four corresponding mouse xenograft models, provided concrete evidence favoring the Cys234 residue of eEF1A1 as the binding site for **33** [130]. Furthermore, it was shown that eEF1A1 plays a significant role in regulating pancreatic cancer cell stemness, its levels positively correlated with pancreatic cancer progression and negatively affecting patient survival.

## 3. Conclusions and Future Perspectives

**Figure 9:**
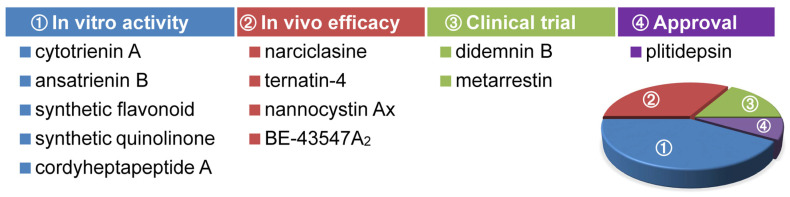
Development status of eEF1A-targeting small-molecule anticancer agents, among which narciclasine, ternatin-4, nannocystin Ax, and BE-43547A_2_ have been validated in at least one in vivo preclinical model, didemnin B and metarrestin have been or currently are being studied in clinical trials, and plitidepsin has gained approval for clinical use. These three groups of compounds are at more advanced development stages than the leftmost group, making up over half of the whole collection of anticancer eEF1A inhibitors discernible from the accompanying pie chart. Their validated efficacy in preclinical models or clinical cohorts lends concrete support to the principle of targeting eEF1A with viable anticancer selectivity.

Translational control with small-molecule agents represents an emerging direction for anticancer drug discovery [132,133,134]. As proofs of principle, hitherto two such drugs have gained approval for clinical use, namely homoharringtonine and plitidepsin. The former is the first protein translation inhibitor for the treatment of chronic myeloid leukemia, which works by fitting into the ribosomal A site so as to block access by the charged tRNA [135]. Pertaining to the present subject, the latter is a specific inhibitor of eEF1A that, at least in part, disrupts its canonical function of assisting translation elongation to combat multiple myeloma [35]. The whole ensemble of known eEF1A-targeting small-molecule agents is compiled in Table 3, alongside a brief summary of their anticancer effectiveness and selectivity. Intriguingly, though sharing the same molecular target, these compounds manifested differential antiproliferative profiles. For example, narciclasine [39], synthetic flavonoids [40], and ternatin-4 [68] within this category displayed preferential anticancer potency against human melanoma, breast carcinoma, and colorectal carcinoma cells, respectively. Presumably, such contrasting selectivity may partially stem from their distinct chemotypes that influence the drug–target interaction as well as the consequent therapeutic outcome in a subtle but profound way, as revealed very recently by Taunton et al. on the inhibitory mode of action of didemnin B and ternatin-4 at the single-molecule level [70]. Overall, the current review outlines the remarkable progress achieved over the recent years in eEF1A-targeting anticancer agents, which are structurally distinct macrocycles and heterocycles, either naturally occurring, developed based on the hit from high-throughput screening, or rationally designed. Their development status is summarized in Figure 9. For the time being, metarrestin is under clinical development as an unprecedented modality for controlling cancer metastasis [42], whereas metarrestin-based PROTACs have also been disclosed for selective degradation of eEF1A2 [108]. Albeit beyond the scope of this review, it is also worth noting that plitidepsin has now entered a clinical trial as a potential anti-SARS-CoV-2 drug [136] since its target eEF1A turned out to be a crucial host protein co-opted by virus to infect human cells [137,138].

In spite of the aforementioned advances, however, there remains much to learn about the exact mechanisms of action of these targeted agents. A more fundamental question lies in the oncogenic mechanism of eEF1A, a multitalented protein capable of both translation elongation and a myriad of moonlighting duties. As evidenced by an illuminating study on plitidepsin [30], it seems indeed viable for malignant cells to exploit certain non-canonical functions of eEF1A for survival. Moreover, the involvement of eEF1A1 in aggressive castration-resistant prostate cancer (CRPC) [139] and non-small cell lung cancer (NSCLC) metastasis [140] has been demonstrated through its complexation with actin and the eEF1A1/MDM2/MTBP signaling axis, respectively. These latest results showcase the potential opportunities of designing next-generation magic bullets [141] that act upon the manifold oncogenic functions of eEF1A, selectively extinguishing the malignant while sparing the normal. It is foreseeable that ongoing investigation of these mechanistic aspects, along with the expanding repertoire of eEF1A-targeting agents, will position us at a better forefront against cancer and other eEF1A-driven diseases as well.

**Table 3:** Summary of eEF1A-targeting small-molecule agents on their anticancer efficacy and selectivity.

Compound	Anticancer Efficacy and Selectivity	Reference
Didemnins	potent against human cancer cell lines from different tissues in vivo validated in several preclinical models didemnin B (**1**) and plitidepsin (**2**) have been studied in multiple clinical trials plitidepsin (**2**, aplidin^®^) has been approved in Australia for combined treatment of relapsed/refractory multiple myeloma with dexamethasone	[36,41,50,51,52,53,54,55,56]
Tamandarins	potent against human cancer cell lines from different tissues	[36,49]
Cytotrienin A (**4**)	potent against human leukemia HL-60 cells (IC_50_ = 7.7 nM) and lung carcinoma A549 cells (IC_50_ = 0.1 μM)	[61,64]
Ansatrienin B (**5**)	potent against three human pancreatic cancer cell lines (IC_50_ range, 0.17–1.69 μM)	[142]
Narciclasine	potent against five human melanoma cell lines (IC_50_~40 nM) in vivo validated in a mice xenograft model of brain metastatic melanoma	[39]
Synthetic flavonoids	potent against human breast cancer cell lines (IC_50_ range, 1–50 μM for MDA-MB231)	[40]
Ternatin-4 (**7**)	potent against human colorectal carcinoma HCT-116 cells (IC_50_ = 4.6 nM) in vivo validated in an aggressive Myc-driven mouse lymphoma model	[68]
Nannocystin A (**11**)	potent against 472 cancer cell lines (IC_50_ range, 5–500 nM) nannocystin Ax in vivo validated in an HCT-116-derived xenograft zebrafish model	[73,91]
Metarrestin (**25**)	excellent antimetastatic selectivity over cytotoxicity (cytotoxicity IC_50_/PNC reduction IC_50_ = 38.3) in vivo validated in several preclinical models currently in a phase I clinical trial for the treatment of metastatic solid tumors	[42,100,101,102,103,104]
Synthetic quinolinones	potent against human cancer cell lines from different tissues (IC_50_ range, 0.56–50 μM)	[110]
Cordyheptapeptide A (**30**)	potent against human colorectal carcinoma HCT-116 cells (IC_50_ = 0.2 μM)	[114]
BE-43547A_2_ (**33**)	potent against human pancreatic carcinoma PANC-1 cells (IC_50_ = 0.87 μM) remarkable hypoxia-selective toxicity against human leukemia K562 cells and breast carcinoma MCF-7 cells (selective index = 28 and 79, respectively) selectively targets pancreatic cancer stem cells (PCSCs) in vivo validated in a pancreatic cancer xenograft mouse model	[127,130]

## Figures and Tables

**Table 1:**
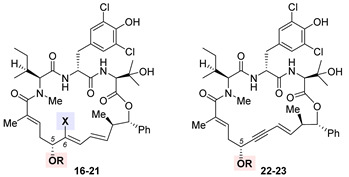
Antiproliferative activity of nannocystin derivatives **16**–**23** against human colorectal carcinoma HCT-116 cells [80].

Compound	X	R	IC_50_ (nM)
Nannocystin Ax (**16**)	Me	Me	0.8
**17**		H	198
**18**	F	Me	1.5
**19**		H	1345
**20**	H	Me	4.3
**21**		H	1549
**22**	---	Me	22.2
**23**		H	1761

**Table 2:** In vitro cytotoxicity of **33** against different types of pancreatic cancer cells (WT, KD, RE-KD, RE-C234S) and its in vivo anticancer efficacy in the corresponding mouse xenograft models [130].

Pancreatic Cancer Cells	In Vitro Cytotoxicity IC_50_ (μM)	In Vivo Tumor Inhibition Rate (%)
WT	1.33	98.8
KD	11.62	20.7
RE-KD	0.80	93.2
RE-C234S	11.61	18.3
